# Combating Ageism with Science: Robert Butler’s Shaping of the National Institute on Aging

**DOI:** 10.1093/geront/gnae167

**Published:** 2024-11-27

**Authors:** Becca R Levy

**Affiliations:** Department of Social and Behavioral Sciences, School of Public Health, Yale University, New Haven, Connecticut, USA

**Keywords:** Activism, Age stereotypes, Ageism, National Institute on Aging, Robert Butler

## Abstract

The physician, scholar, and activist Robert Butler devoted much of his life to trying to end ageism in order to create a society that provides older persons with equal rights and opportunities. His passion for fighting ageism led to his becoming the founding director of the National Institute of Aging (NIA) and set the stage for many of its achievements during the past 50 years. This article explores how Butler first became committed to overcoming ageism, how he made a strong case for setting up NIA as a headquarters to combat ageism with science, and how he launched NIA as a multidisciplinary organization that could draw on research, training, and public policy as weapons against ageism. Finally, this article highlights how Butler, through his anti-ageism activities in later life, personified the possibilities he had done so much to make available to older persons through his launch of NIA.

It is increasingly within our power to intervene directly in processes of aging, with prevention, treatment, and rehabilitation…. If, however, we fail to alter present negative imagery, stereotypes, myths, and distortions concerning aging and the aged in society, our ability to exercise these new possibilities will remain sharply curtailed. Butler, 1989, p.139

As Robert Butler began his comments by thanking everyone for attending his 80th birthday party, the gentleman beside me whispered, “Do you know Butler is a lamed vavnik?” After investigating this term following the party, I learned it referred to an ancient mystical belief that 36 angels are placed on the earth to do good works. This seemed fitting because Butler had an otherworldly ability to identify and address a problem that had been overlooked by others, even though its affliction of older persons was widespread. He gave this problem a name, “ageism,” and defined it as “systematic stereotyping and discrimination against people simply because they are old” (Butler, 2008, p. 40).

Butler devoted much of his life to battling ageism with the goal of helping to create a society in which older persons could age with equal rights and opportunities. This passion for eliminating ageism led to his becoming the founding director of the National Institute of Aging (NIA) and set the stage for its accomplishments in research, training, and advocacy for older persons during the past 50 years.

## Origin of Butler’s Determination to Overcome Ageism

Butler’s respect for aging arose from being raised by his grandparents, who were hardworking chicken farmers. He was particularly impressed by his grandfather’s care of sick chickens, which inspired the young Butler’s interest in the field of medicine (Butler, 1975). Despite a series of calamities, which included the sudden death of Butler’s grandfather, the loss of the family’s savings and farm during the Depression, and the move to a hotel that burned down with all their possessions, Butler wrote, “What I remember even more than the hardships of those years was my grandmother’s triumphant spirit and determination” (Butler, 1975, p. x).

In contrast to these recollections of his grandparents’ strengths, when Butler attended medical school, he was shocked to hear his professors refer to older people with “a variety of words in the cruel medical lexicon, generally unknown to the public, that reflect negative attitudes toward patients, especially older patients: ‘crock’ to describe older people and middle-aged women, ‘gork’ (god only really knows), ‘gomer’ (for get out of my emergency room), ‘vegetable,’ and ‘SPOS’ (for Special Piece of S–-)” (Butler, 2005, p. 84). Butler recalled that in medical school, “I first became conscious of prejudice toward age” (Butler, 1989, p. 140). The training he received reinforced this: “In medical school, every effort was made to introduce us to middle-aged and younger patients, but older patients were considered to be beyond help—in other words, not teaching material” (Pincock, 2010, p. 588). Further, “When we did see older patients, we saw them as archives, museums of pathology” (Columbia School of Public Health, 2024).

One night, during his medical internship, when Butler was working his hospital shift, he gave a 72-year-old anxious patient a sedative to calm him down, but, instead, the man became even more agitated and was transferred to a psychiatric hospital. Afterward, Butler searched the literature to try to understand the patient’s response. He learned that the medication he prescribed can have the opposite effect on older patients, although “this lesson was never taught” (Butler, 2005, p. 84).

These medical school experiences sensitized Butler to ageism. They also started him on a path to combat this form of injustice when launching NIA.

## Butler Makes Case for Establishing NIA as a Way to Fight Ageism

Butler’s writing about ageism in two books made a strong case for the creation of an NIA. A premise of both books was that “Concerning the treatment of ageism as a disease, I find that knowledge is the most basic intervention, serving as an antidote for numerous and erroneous but widely held beliefs” (Butler, 1989, p. 138).

The case for setting up an NIA was made indirectly through his first book, *Human Aging: A Biological and Behavioral Study*, which resulted from a job Butler took soon after medical school. It involved intensely studying older persons with an interdisciplinary team of psychologists and biologists. He explained that “some of my old friends and colleagues questioned the wisdom and logic of my choice, and one jokingly called me a necrophiliac” (Butler, 2005, p. 84). Undeterred, Butler continued with the team whose purpose was to disaggregate the effects of disease from normal aging (Birren et al., 1963). Previous aging research was mainly conducted on the small percentage of older persons living in nursing homes and focused on those with chronic diseases (Birren et al., 1963; Pincock, 2010). Therefore, Butler explained, “We did not know what robust, healthy older people should be like until we undertook the first comprehensive studies of this population” (Glaser, 2008, p. 978). Their research contradicted a number of negative age stereotypes. For example, Butler wrote that his work with the team “led me to the conclusion that senility was not inevitable with aging but due to specific causes” (Butler, 1999, p. 390).

Butler made his case for an NIA directly in a second book, *Why Survive? Being Old in America*. In it he laid out the harm and ubiquitousness of ageism: “The tragedy of old age is not the fact that each of us must grow old and die but that the process of doing so has been made unnecessarily and at times excruciatingly painful, humiliating, debilitating, and isolating through insensitivity, ignorance and poverty” (Butler, 1975, pp. 2–3). He concluded that ageism needed to be addressed by the creation of a “National Institute on Aging,” which would be able to “coordinate studies in the biomedical, social, and behavioral aspects of the aging process and to conduct and support education and training in both research and service in the field of aging” (Butler, 1975, p. 355).

## Butler Overcame Ageism to Launch the NIA

When Butler was lobbying for the establishment of NIA, he faced ageist opposition from scientists and government officials who considered aging to be not worth studying because, they asserted, it is a time of inevitable decline, and older persons are undeserving of the targeted investment of resources and attention (Butler, 1969, 1975; Glaser, 2008). He saw an additional obstacle: “since the attitudes of the members of a society shape the policies that govern it, bias, prejudice, and stereotypes can interfere with effective policy formulation” (Butler, 1980, p. 8). Butler lamented that although an urgent need existed to set up an NIA, the government was “dragging its feet in getting the Institute started” (Butler, 1975, p. 355).

To counter the opposition, he maintained that battling ageism through supporting the aging research of an NIA can benefit us all, both individually and as a society: “When we talk about old age, each of us is talking about his or her own future. We must ask ourselves if we are willing to settle for mere survival when so much more is possible” (Butler, 1975, p. xiii). In addition, he contended that “The quality of a culture can be measured by its regard for its least powerful members, for example, its care for the elderly” (Butler, 1969, p. 245).

Butler’s arguments were persuasive in overcoming the opposition to establishing an NIA. Congress mandated its creation and appointed him its first director. When he launched NIA, Butler outlined a vision that continues to shape the Institute 50 years later (Butler, 1977; Harper, 2024). This comprised forming the first governmental organization to include a focus on combating ageism with science through research, training, and advocacy, and, thereby, improving the lives of older persons.

## NIA Research as Butler’s Weapon Against Ageism

In his NIA mission statement, Butler stressed the importance of research that examined “the reasons and remedies for the stigma attached to old age” (Butler, 1977, p. 103). He believed that aging research, even when not focused on age stigma, can also reduce ageism. Such research could replace negative “stereotypes, myths, and images of old age” with “more factual information” (Butler, 1993, p. 53).

When the NIA was established, many who considered aging to be purely a time of biological decline thought the Institute should concentrate exclusively on disease, using a biomedical approach (Achembaum, 2013; Butler, 1975, 2008). Whereas, Butler insisted that for NIA to oppose ageism and promote productive aging, it was important to also focus on healthy aging, which he called “the neglected stepchild of the human life cycle” (Butler, 1975, p. 1).

In addition, Butler explained that for aging research to best address ageism, it should be “basic, meticulous and rigorous” as well as multidisciplinary (Butler, 1977, p. 99). His NIA mission statement also made the point that “For all too long the biological and physical scientists have thought self-righteously of themselves as being basic scientists, with the implication that the ‘softer’ scientists are somehow not basic. This reflects a narrow value system….Indeed, the problems that exist between one person and another, or between one people and another, are about as basic a body of questions as one could ask” (Butler, 1977, p. 99). Butler enforced these priorities when he established both the intramural research branch that conducted research on NIA’s campus and the extramural branch that managed grants to investigators beyond the campus.

Soon after becoming the NIA director, Butler learned that his book documenting ageism, *Why Survive? Being Old in America,* had been awarded the Pulitzer Prize. He believed this served as a credential that enabled him to work with other National Institutes of Health (NIH) directors who were initially skeptical about the need for an Institute devoted to aging (Columbia School of Public Health, 2024). During his time as director, Butler managed to set up research projects with all the NIH institutes, effectively increasing the capacity of the NIA to counter ageism by drawing on a greater array of resources (Columbia School of Public Health, 2024). His success at collaborating with other NIH institutes, as well as with other government agencies, and with making NIA-funded research multidisciplinary continues today (Administration for Community Living, 2024; Kelley et al., 2024).

## NIA Training as Butler’s Weapon Against Ageism

Another area that Butler made an NIA priority, with an eye on combating ageism, was training for those interested in careers as healthcare providers. He invited the deans of all the medical schools in the United States to the NIH campus to discuss how to best incorporate geriatrics into their curricula (Achembaum, 2013). Butler believed it was important for geriatric training to counter negative stereotypes about aging, such as the belief that disease in later life is always irreversible. More generally, he wanted to help overcome the “unconscious attitudes on the part of the physicians [that] may lead them to feel that treating the complex physical problems of older people is not worth their time” (Butler, 1980, p. 9).

Butler was convinced that in addition to improving the training of clinicians, NIA should prioritize confronting ageism by providing training for careers in aging research (Butler, 1996). In his first year at NIA, he funded 10 trainees; in his second year, he increased the funding to 90 (Butler, 1996). NIA continues this commitment by currently funding over 1000 trainees through fellowships and numerous programs—including one named after Butler for postdoctoral fellows and junior faculty conducting studies of aging (Harper, 2024).

I feel very fortunate to have directly benefited from the training fellowships that Butler established. The NIA National Research Service Award postdoctoral fellowship allowed me to design and carry out a series of studies investigating the effect of ageism—specifically, negative age stereotypes. In one of the experiments, I found that when older persons are exposed to these stereotypes, their cardiovascular reactivity to stress increases; when this occurs chronically, it can lead to worse cardiovascular health (Levy et al., 2000). I was honored that Butler cited this work in an article he wrote, entitled “Combating Ageism,” as evidence of the “adverse physiological effects of ageism” (Butler, 2009, p. 211).

## Public Policy as Butler’s Weapon Against Ageism

In addition to research and training, Butler saw public policy as crucial to overcoming ageism. He believed the “government needs to be involved in all levels” to bring about social change (Butler, 1989, p. 147).

In his NIA mission statement, Butler identified the types of ageism that he thought should be targeted by policy reform. These included mandatory retirement. He argued it needed to be abolished “in order for the NIA to assist old people in remaining independent and contributing members of society” (Butler, 1977, p. 103). At the time, 4 million of the 13 million retired persons in the United States over the age of 65 had been forced to retire because of their age (Sanjek, 2009).

Butler worked closely with the octogenarian Congressman Claude Pepper to expand coverage of the Age Discrimination in Employment Act so that it virtually ended mandatory retirement (Butler, 2005). The United States was the first country to do so. In his NIA office and in his subsequent offices, Butler displayed a photograph of himself with Congressman Pepper celebrating their legislative victory.

Congressional hearings provided Butler with a platform to advocate for public policies to remedy various forms of ageism. On one of these occasions, I joined him in testifying before the United States Senate Special Committee on Aging, which was considering whether legislation was needed to address ageism in media and marketing. Butler’s statement centered on the published images of older persons, which ranged from denigrating to empowering. To illustrate, he displayed contrasting representations: a crowd of menacing older persons on a magazine cover with the headline “Greedy Geezers,” compared with a vibrant 92-year-old woman from the book *Wise Women: A Celebration of their Strengths Courage and Beauty* by his friend, the photographer Joyce Tenneson (Tenneson, 2002). To demonstrate the potential effect of aging images, in my testimony I presented an example of my research, conducted with the support of an NIA K01 Award, that showed older individuals who had assimilated positive age stereotypes from society tended to live, on average, seven-and-a-half-years longer than their peers who had assimilated negative age stereotypes from society (Levy et al., 2002).

Butler thought policy should address the tendency for ageism to become even more harmful when it is combined with other forms of prejudice. He initiated NIA workshops, intended to generate requests for proposals that included this intersectionality. Among these workshops was one that focused on older women because he recognized that ageism was even more destructive when compounded by sexism (Achembaum, 2013; Butler, 1977, 2008).

Another policy objective that Butler promoted was ending the exclusion of older persons from clinical trials (Butler, 1977, 2008). Although he was able to increase the number of clinical trials that included older participants during his time at the head of NIA, older persons remain underrepresented, even in those trials that target conditions that are most common among older persons (Levy, 2022). Progress towards Butler’s goal was recently implemented: NIA spearheaded the adoption of a new policy by NIH that requires researchers applying for NIH funding of projects, including clinical trials, to explain how they plan to “include individuals of all ages unless there is a scientific or ethical reason for exclusion” (Harper, 2024).

## Butler’s Later-Life Anti-Ageism Activism

After leaving NIA, Butler continued his anti-ageism activities past the age that once determined mandatory retirement. Appropriately, he carried out many of these activities in a New York City townhouse that had previously served as the offices of the psychologists Kenneth and Mimi Clark, who, like Butler, used science to confront bigotry. The Clarks’ research provided the rationale for United States versus Topeka Board of Education, the United States Supreme Court decision that undermined racism in the form of school segregation. When Butler discovered this link to the Clarks, he had a large plaque commemorating their work installed at the townhouse’s entrance.

I first met Butler when he was 73 and serving, among his other positions, as the program chair for the Brookdale Fellowship for Leadership in Aging, continuing the tradition he began at NIA of supporting gerontology training. Soon after completing graduate school, I was elated to be awarded a Brookdale fellowship. My excitement grew when I learned that Butler would be my fellowship advisor. After all, he was the person who, through his writing, inspired me to pursue a career in aging research and to seek ways to address ageism. He reinforced these goals when he invited me to work with him on several of his anti-ageism projects.

As an example of these projects, also at the age of 73, Butler began his Age Boom Academy (he explained to me that he selected this name as a contrast to the infantilizing term “baby boom” generation). He aimed for the Academy to train journalists to become mindful of ageism and the need to expose it.

At the age of 75, Butler produced a Declaration of Human Rights for Older Persons, which was intended to counter ageism throughout the world. He stated in the Declaration that older members of society are “often treated in cruel and inaccurate ways in language, images and actions” and “are not provided the same rich opportunities for social, cultural and productive roles and are subject to selective discrimination in the delivery of services otherwise available to other members of the society” (Butler, 2002, p. 152). He presented the Declaration to the United Nations World Assembly of Ageing in Madrid, where it was approved by 160 countries (Achembaum, 2013; Glaser, 2008).

At the age of 81, Butler published *Longevity Revolution: The Benefits and Challenges of Living a Longer Life.* In this book, he pointed out that “The NIA’s budget is less than 3% of the total NIH budget…despite the revolution in longevity that has occurred and is likely to continue to occur” (Butler, 2008, p. 105). To emphasize the value of increased NIA funding, he explained that “Heavy investment in biomedical, behavioral, and social research is the ultimate cost containment, the ultimate disease prevention, and the ultimate service…[and it] will also do much to overcome many of the problems of age, as well as ageism” (Butler, 2008, p. 54).

Butler’s executive assistant, Morriseen Barmore, who worked closely with him during the last two decades of his life, recalls that while directing NIA and in the years that followed, he “fought against ageism every single day” (Barmore, 2024). Butler’s later-life activities were not only significant in their own right; they also countered ageism by demonstrating that these years can be a time of impressive contributions. That is, he personified the possibilities he had done so much to make available to older persons through his creation and leadership of NIA.

## Data Availability

This article does not report data; therefore the preregistration and data availability requirements are not applicable.
